# The Cell Biology of *Leishmania*: How to Teach Using Animations

**DOI:** 10.1371/journal.ppat.1003594

**Published:** 2013-10-10

**Authors:** Dirceu E. Teixeira, Marlene Benchimol, Juliany C. F. Rodrigues, Paulo H. Crepaldi, Paulo F. P. Pimenta, Wanderley de Souza

**Affiliations:** 1 Instituto de Bioquímica Médica, UFRJ, Rio de Janeiro, Brasil; 2 Fundação Cecierj/CEDERJ, Rio de Janeiro, Brasil; 3 Instituto Nacional de Metrologia, Qualidade e Tecnologia, Inmetro, Rio de Janeiro, Brasil; 4 Universidade Santa Úrsula, Rio de Janeiro, Brasil; 5 Instituto de Biofísica Carlos Chagas Filho, UFRJ, Rio de Janeiro, Brasil; 6 Núcleo Multidisciplinar de Pesquisa UFRJ-Xerém, UFRJ, Rio de Janeiro, Brasil; 7 Instituto Nacional de Ciência e Tecnologia de Biologia Estrutural e Bioimagem, Rio de Janeiro, Brasil; 8 Centro de Pesquisas René Rachou, FIOCRUZ, Minas Gerais, Brasil; University of Wisconsin Medical School, United States of America

Parasitic protozoa are important agents of human and animal diseases in Brazil and around the world. Protozoan parasites of the *Leishmania* genus are the causative agent of leishmaniasis, one of the most important neglected tropical diseases as designated by the World Health Organization (WHO). Leishmaniasis affects about 12 million people worldwide and can be divided into the following three main clinical manifestations: cutaneous, mucocutaneous, and visceral leishmaniasis [1].

## Didactic View Using 3D Animations of *Leishmania* Life Cycle

*Leishmania* parasites have a complex life cycle that involves both vertebrate and invertebrate hosts and two developmental stages: promastigotes, the proliferative form found in the lumen of the female sandfly, and amastigotes, the proliferative form found inside several types of mammalian host cells. The life cycle of this parasite is taught at varying levels of education ranging from secondary education to graduate school. The current teaching method used to convey the concepts associated with this life cycle is based on formal lectures using classic materials with little emphasis on the use of three-dimensional (3D) animation models. In this report, we present a new instructional approach with modern schemes and dynamic models that include 3D animations; this approach is based on the information presently available on the *Leishmania* life cycle, most of which has been obtained using modern microscopy techniques. As an instructional tool, the proposed animations are more effective than the static graphics when teaching dynamic events [2], [3]. Studies in biology courses have shown that 3D animations lead to increased student understanding and retention of cell biology information [4].

**Figure 1:**
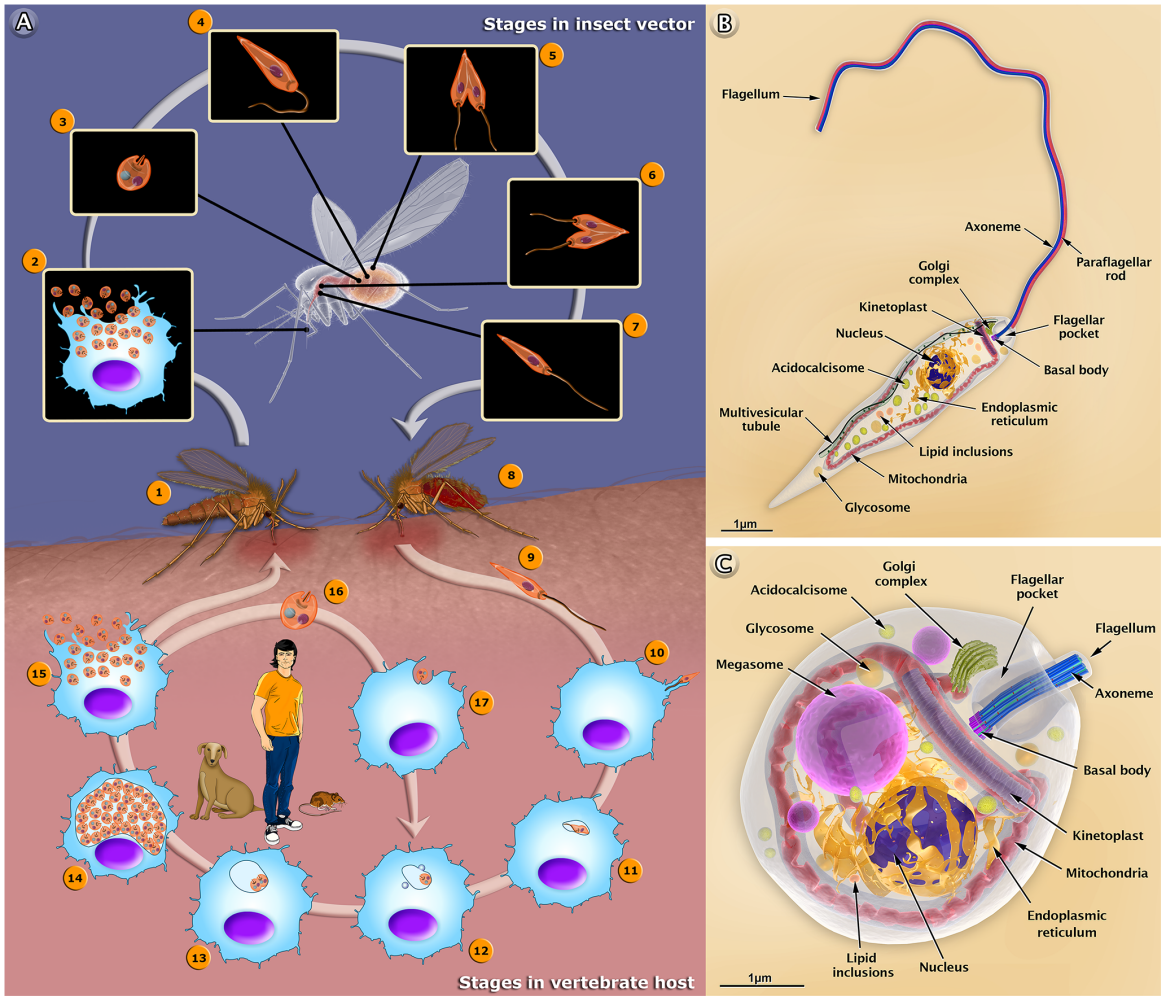
The life cycle of *Leishmania amazonensis* (A), and structural organization of the promastigote (B) and amastigote (C) forms. (A) The female sandfly (1) insect bites an infected mammal during the blood meal. Infected macrophages (2) with amastigote forms. (3) Amastigote form. (4) Amastigotes transform into procyclic promastigotes. (5) Procyclic promastigotes multiply in the midgut. (6) Promastigotes migrate toward the stomodeal valve in the anterior midgut and re-initiate cell division. (7) Promastigotes transform into infective metacyclic promastigotes. (8) The female sandfly releases the metacyclic promastigotes into a new mammalian host via regurgitation during the blood meal. (9) Metacyclic promastigotes. (10) Metacyclic promastigotes infect macrophages. (11) Metacyclic promastigotes transform into amastigotes. (12) Amastigotes attach to the membrane of the parasitophorous vacuole. (13) Amastigotes multiply in the vacuole. (14) Intense amastigote multiplication. (15) Amastigotes burst out of the cell. (16) Amastigote form. (17) An amastigote infects a macrophage. In the central portion of the figure, we added the most important reservoirs involved in the maintenance of the parasite. Schematic 3D representations of the organelles found in the *Leishmania* promastigote (B) and amastigote (C).

The life cycle of *Leishmania* involves two different hosts: a female sandfly and mammals (including humans and dogs), as summarized in Figure 1A. Herein, we used *Leishmania amazonensis*—an important species in Brazil—as a model to describe the basic aspects of the interaction with host cells. It is important to point out that the amastigotes of this protozoan live in large cytoplasmic vacuoles and attach to the membrane lining the parasitophorous vacuoles. Other species live in tight vacuoles and are randomly distributed within the vacuoles. We also produced a 3D video that shows the basic aspects of the life cycle of *Leishmania* in the human host (http://www.inbeb.org.br/conteudo.asp?idsecao=313) and inside the female sandfly (http://www.inbeb.org.br/conteudo.asp?idsecao=314).

## Different Forms of the *Leishmania* Parasite: Drawings and Animations

The 3D organization of the two developmental stages, the promastigote and amastigote forms of *Leishmania*, and the presence and distribution of their structures and organelles are illustrated in Figures 1B and C. Organelles such as the nucleus, the endoplasmic reticulum, the kinetoplast-mitochondrion complex, the Golgi complex, the acidocalcisomes, and the components of the endocytic pathway are indicated. These images were created based on micrographs obtained by transmission electron microscopy. We also produced videos that show detailed 3D animations of the structural organization of the promastigote (http://www.inbeb.org.br/conteudo.asp?idsecao=312) and amastigote (http://www.inbeb.org.br/conteudo.asp?idsecao=311) forms.

## Animations of the Interaction of *Leishmania amazonensis* with Mammalian Host Cells

**Figure 2:**
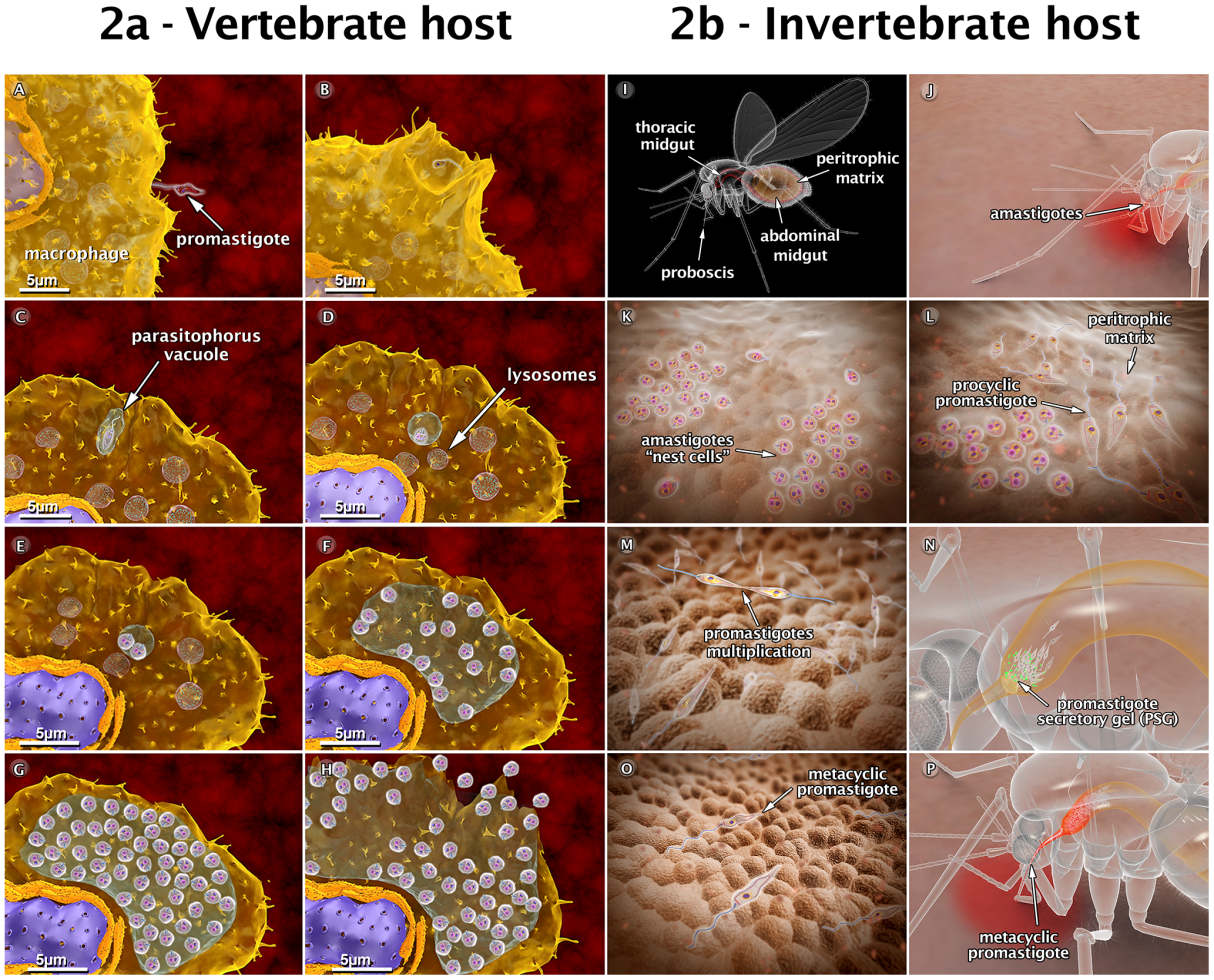
Schematic 3D view of the phases of interaction between the *Leishmania amazonensis* parasite and vertebrate cells (2a), and between the parasite and the sandfly (2b). (A) Attachment of a promastigote to the macrophage surface. (B) The process of internalization via phagocytosis begins with the formation of pseudopods (C), leading to the formation of the parasitophorous vacuole (PV). In the PV, the promastigote transforms into an amastigote. (D) Recruitment and fusion of host cell lysosomes with the PV takes place. (E) In the PV, amastigotes divide several times. (F–G) Intense multiplication generates several hundreds of amastigotes. (H) The host cell bursts, and the parasites reach the extracellular space. (I) Schematic view of female sandfly showing the digestive tract. (J) During a blood meal, a female sandfly ingests infected macrophages with amastigote forms present in the blood of the vertebrate host. (K) Amastigotes form “nest cells” in the abdominal midgut. (L) Amastigotes transform into procyclic promastigotes. (M) Promastigotes multiply and attach to the midgut epithelium. (N) Parasites migrate toward the anterior midgut, resume replication and start to produce promastigote secretory gel (PSG). (O) Promastigotes transform into infective metacyclic promastigotes. (P) Metacyclic promastigotes infect a new mammalian host via regurgitation during the blood meal. These images are based on micrographs obtained by scanning and transmission electron microscopy and by video microscopy.

The infection begins when a phlebotomine sandfly harboring *Leishmania* protozoa bites a human or other mammalian host for blood feeding. During the bite, the insect injects saliva that prevents blood clotting [5]. Following the ingestion of blood, metacyclic promastigotes are released and enter into the host skin via regurgitation [6]. A simple bite releases many substances that induce rapid infiltration of neutrophils and substantial recruitment of macrophages into the skin [7]. The parasites reach the mammalian skin and first invade the neutrophils, which are rapidly recruited to the bite site and macrophages. However, other cell types, such as Langerhans cells and fibroblasts, can also be infected [7]. This first step of interaction with host cells is exemplified in the present study using neutrophils and macrophages. Both involve recognition and adhesion, which is followed by signaling and invasion [8]. Neutrophils are thought to play an important role, acting as a “Trojan horse” [7], while macrophages are important for the final establishment and amplification of the infection. The parasite attaches to the host cell surface via either the flagellum or the cell body. This adhesion involves the recognition of molecules exposed on the parasite's surface, such as lipophosphoglycans (LPGs) [6] and the gp63 glycoprotein [9]. These molecules bind to different receptors found on the surface of the macrophages, including complement receptors (CR1 and CR3), mannose receptors (MRs), and fibronectin receptors (FnRs) [10]. The process of internalization via phagocytosis begins with the formation of pseudopods. Thus, the parasite attaches to the macrophage surface and is then internalized into a vacuole known as the parasitophorous vacuole (PV). In the PV, the metacyclic promastigote transforms into an amastigote; this is followed by fusion of the host cell lysosomes with the PV [11]. Some of the amastigotes become attached to the membrane of the vacuole, while others remain free in the vacuole and begin to proliferate, dividing multiple times. Following an intense multiplication, the macrophage membrane ruptures, thereby releasing the amastigotes into the tissue; these amastigotes can invade new macrophages or be ingested by a new female phlebotomine during its blood meal. It is important to point out that during the infection with *L. amazonensis*, the amastigotes multiply inside a very large PV, which contains many parasites attached to the membrane of the vacuole. In other species, including all Old World strains as well all *Viannia* species, the amastigotes remain segregated within their own small, tight PV following each division. Figure 2A–2H summarizes the steps of the biological cycle of *Leishmania amazonensis* in the vertebrate host cell, but this cycle is better visualized in a 3D video (http://www.inbeb.org.br/conteudo.asp?idsecao=316).

## Animations of the Behavior of *Leishmania* in the Insect Vector

The infection of the invertebrate host begins when a female sandfly insect bites an infected mammal during its blood meal. The female uses its mouthparts to pierce the skin, lacerating capillaries and forming a hemorrhagic pool; from this, it ingests blood containing macrophages infected with amastigotes [12]. The blood meal is digested in the abdominal midgut of the insect. In this new environment, amastigotes are grouped together to form clusters or “nest cells,” which are enclosed by a bag-like structure called the peritrophic matrix (PM) that surrounds the blood meal and protects it from digestive enzymes [13], [14]. The amastigotes then transform into a replicative form called procyclic promastigotes. During the transformation, intermediate forms between the amastigotes and promastigotes are more susceptible to death by digestive enzymes found in the intestinal environment [13]. Subsequently, the anterior portion of the PM breaks down, and the parasites are released into the midgut epithelium of the insect. Promastigotes divide via binary fission and become attached to the microvilli of the midgut epithelium. This adhesion occurs predominantly throughout the region of the flagellum and involves the participation of an LPG exposed on the promastigote's surface [15]. When the parasites detach from the epithelium, the promastigotes migrate toward the stomodeal valve located in the anterior midgut, where they concentrate and reinitiate cell division [16]. These are responsible for the production and secretion of a gel (PSG) that acts as a plug obstructing the midgut and pharynx [17], [18]. During the production of the PSG, the parasites start to transform into infective metacyclic promastigote forms. This differentiation process is called metacyclogenesis. Parasites cause damage to the stomodeal valve, thereby interfering with its function and facilitating a reflux of the parasites from the thoracic midgut [19]. Consequently, during subsequent insect bites, infective metacyclic promastigotes are released and can thus infect a new mammalian host, thereby starting the cycle over. Figure 2I–2P summarizes the steps of the biological cycle of *Leishmania* in the invertebrate host, but this can be better visualized in a 3D video (http://www.inbeb.org.br/conteudo.asp?idsecao=314).

## Conclusions

Animations are powerful tools that can quickly and easily communicate scientific ideas that may be difficult to understand when described only using words or static images. Together, the 3D schemes and the dynamic 3D videos allow a better visualization of the *Leishmania* developmental stages, which include several dynamic cellular processes and the interaction between the protozoan and the vertebrate and invertebrate hosts.

The multimedia materials described herein can be used to present a comprehensive view of the protozoan life cycle to students. These materials also offer dynamic models that improve our understanding of some important biological processes. The proposed multimedia material is useful for a broad audience, including students, teachers, and any member of the general public who may be interested in parasites.
